# Enhanced Ni(II) Removal from Wastewater Using Novel Molecular Sieve-Based Composites

**DOI:** 10.3390/ma17133211

**Published:** 2024-07-01

**Authors:** Zengjie Li, Yalin Lei, Li Dong, Li Yu, Cong Yin

**Affiliations:** 1 Department of Safety Supervision and Management, Chongqing Vocational Institute of Satety Technology, Chongqing 401331, China; 15922535934@163.com; 2 College of Chemistry and Chemical Engineering, Chongqing University of Science and Technology, Chongqing 401331, China; 18503421833@163.com (Y.L.); dongyyds_04@163.com (L.D.); 3 Xi’an Research Institute of Hi-Tech, Xi’an 710025, China

**Keywords:** Ni(II), removal, molecular sieve, adsorption

## Abstract

This study focuses on the efficient removal of Ni(II) from spent lithium-ion batteries (LIBs) to support environmental conservation and sustainable resource management. A composite material, known as molecular sieve (MS)-based metal–organic framework (MOF) composites (MMCs), consisting of a synthesized MS matrix with integrated MOFs, was developed for the adsorption of Ni(II). The structural and performance characteristics of the MMCs were evaluated using X-ray diffraction (XRD), Fourier transform infrared spectroscopy (FT-IR), scanning electron microscopy (SEM), and N_2_ adsorption–desorption isotherms (BET). Batch adsorption experiments were conducted to assess the Ni(II) adsorption performance of the MMCs. The results revealed that, under conditions of pH 8 and a temperature of 298 K, the MMCs achieved near-equilibrium Ni(II) adsorption within 6 h, with a maximum theoretical adsorption capacity of 204.1 mg/g. Further analysis of the adsorption data confirmed that the adsorption process followed a pseudo-second-order kinetic model and Langmuir isotherm model, indicating a spontaneous, endothermic chemical adsorption mechanism. Importantly, the MMCs exhibited superior Ni(II) adsorption compared to the MS. This study provides valuable insights into the effective recovery and recycling of Ni(II) from spent LIBs, emphasizing its significance for environmental sustainability and resource circularity.

## 1. Introduction

Nickel is an important non-ferrous metal with a wide range of applications in the electronics industry and battery production [1,2]. Especially in the production of ternary lithium batteries, nickel, as one of the main components of the positive electrode material, is widely used [3]. However, with the rapid development of the global electric vehicle market and the increasing demand for electronic products, nickel resources are facing increasingly severe supply pressures. According to statistics, the current global nickel reserves are limited, and nickel mining and production also face many problems, such as high costs, environmental impacts, etc., making the rational utilization of nickel resources particularly important [4,5].

Spent lithium-ion batteries (LIBs) contain a large amount of nickel [6]. If these nickels are not effectively recovered and reused, it will not only lead to a waste of resources but also pose significant pollution risks to the environment [7]. The treatment and disposal of spent LIBs often face many challenges, such as the difficulty of heavy metal recovery, potential threats to the surrounding environment and human health, etc. [2,8]. Therefore, it is crucial to develop efficient and economical techniques to effectively separate and recover nickel elements from spent LIBs. This can not only effectively solve the problem of waste battery treatment and disposal but also achieve resource recycling and reuse, achieving the dual purposes of resource recycling and environmental protection. 

The separation of metal ions is a key issue in the fields of environmental protection and resource recovery, and adsorption technology, as a commonly used separation method, has significant advantages in separating metal ions [4]. Adsorption technology is characterized by simple operation, high efficiency, rapidity, and broad adsorption capabilities for various substances, making it widely used in the separation and enrichment processes of metal ions [9]. In addition to adsorption, methods for separating metal ions also include solvent extraction [10], membrane separation [11], precipitation, etc., but relatively speaking, adsorption is more emphasized in practical applications.

Common adsorption materials at present include carbon materials, biological materials, etc. Carbon materials such as activated carbon, carbon nanotubes, etc., with high surface area and pore structure, exhibit excellent performance in the adsorption of metal ions. Biological materials such as biopolymers, microorganisms, plant extracts, etc., possess natural adsorption properties and have a certain potential for the biological adsorption of metal ions. However, existing adsorption materials still have some drawbacks, such as limited adsorption capacity, low selectivity for specific metal ions, poor regenerability, etc., which restrict their application in the separation of metal ions.

Therefore, there is an urgent need to develop new adsorption materials to address these issues. Modified molecular sieves (MSs) are one of the important directions in current research [12]. The purpose of modifying MS is to enhance their adsorption performance, further expanding their application fields. Modification can be achieved by introducing new functional groups or coordinating atoms, controlling their crystal structure and pore structure, or improving their surface affinity [13,14]. These modification methods can significantly improve the adsorption capacity, selectivity, and regenerability of MSs, making them more suitable for various environmental governances, resource recoveries, and chemical industries [15,16].

In recent years, researchers have actively explored various new methods of modifying MSs, such as introducing functional groups, surface modification, synthesizing composite materials, etc. [17]. Among them, the synthesis of composite materials is an effective way. By combining MSs with other functional materials, composite materials with synergistic effects can be formed, overcoming the shortcomings of traditional MSs and endowing them with new properties [18]. This method not only enhances the adsorption performance and stability of MSs but also broadens their application range in different fields.

Therefore, modifying MSs to enhance their adsorption performance and functionalization has become one of the hotspots in current research. By continuously exploring new modification methods and material combinations, more efficient and environmentally friendly MS materials can be developed, making greater contributions to environmental protection, resource recovery, and the development of the chemical industry [12,13,16].

Metal–organic frameworks (MOFs) as a new type of material have shown great potential in the field of metal ion separation [19,20,21,22]. MOFs have a high specific surface area, controllable pore structure, and abundant functional groups, making them perform well in adsorption, separation, storage, etc. Especially in the process of metal ion separation, MOFs exhibit excellent adsorption performance and selectivity, providing new possibilities for solving the problems of metal ion separation and enrichment [23,24]. However, due to the relatively high cost of MOF preparation, its application range and promotion in practical applications are limited.

To overcome the problem of high cost in preparing MOFs and further leverage their advantages in metal ion separation, we propose a reasonable combination of MOFs with MSs. This combination can fully utilize the efficient adsorption performance of MOFs and the structural stability of MSs, complementing each other’s shortcomings, and achieving a more efficient metal ion separation process. By designing and constructing MOFs and MS composite materials reasonably, the advantages of both can be combined to increase the adsorption capacity and stability of the materials, thereby achieving effective separation and enrichment of metal ions.

In this study, we synthesized a composite material called MS-MOFs composites (MMCs), which consisted of a molecular sieve (MS) with aluminum ions as metal ion sources and added organic ligands of 1,3,5-benzenetricarboxylic acid (H3BDCH3BTC). This novel material was utilized for the separation of nickel ions, offering a promising solution for the depletion of nickel resources and the treatment of spent LIBs. The new composite material not only reduces the preparation cost of MOFs but also enhances its adsorption capacity for nickel ions. The development of these innovative composite materials provides a novel approach towards environmental protection and resource utilization, with significant practical and scientific implications.

## 2. Experimental Section

### 2.1. Materials

NaAlO_2_, SiO_2_ particle, and 1,3,5-benzenetricarboxylic acid (H_3_BTC) were obtained from Aladdin Reagent, Shanghai. N,N-dimethylformamide (DMF, 99.8%), ethanol, HCl, and NaOH were purchased from Chengdu KeLong Chemical Reagent Factory (Chengdu, China). All reagents were used without further purification.

### 2.2. Preparation of MMCs

In a 250 mL flask, 2 g of SiO_2_ were combined with a 30% NaOH solution and refluxed at 90 °C for 4 h. Around 2.7 g of NaAlO_2_ and deionized water were subsequently added. The mixture was stirred and refluxed for 24 h at a high temperature of 120 °C for crystallization. After reaching room temperature, the product was filtered and washed with deionized water until the pH of the filtrate dropped below 11. The resulting residue was dried overnight at 120 °C, yielding a white powder identified as the MS. Next, 2.00 g of the MS and 1.2 g of H_3_BTC were added to 100 mL of N,N-dimethylformamide in a reflux setup at 120 °C for 12 h. The solid product obtained underwent centrifugation, followed by three rinses with DMF and ethanol. It was then vacuum-dried overnight at 60 °C to yield the final solid product, MMCs.

### 2.3. Characterization of MMCs

Scanning electron microscope (SEM) analysis was performed using a JSM-5610LV microscope (STARJOY LIMITED, Tokyo, Japan). X-ray diffraction (XRD) was conducted by scanning from 5° to 60° with an XRD-7000 instrument (CuKα radiation at 40 kV and 30 mA, Shimadzu, Kyoto, Japan). Fourier transform infrared (FT-IR) spectra of the samples were obtained using a Fourier transform infrared spectrometer (Thermo Fisher, Waltham, MA, USA). The BET surface area was determined by N_2_ adsorption–desorption (Autosorb-iQ, Quantachrome, Boynton Beach, FL, USA).

### 2.4. Adsorption Experiment

Take 10 mg of the adsorbents MS and MMCs and place them separately in 50 mL simulated wastewater solutions with 10 mg/L of Ni(II), adjusting the pH using a 0.1 mol/L NaOH or HCl solution. After a specific duration of oscillation in a constant temperature shaking incubator, transfer a specific volume of the supernatant to a centrifuge tube, centrifuge at 10,000 rpm for 10 min, then extract a sample and determine the concentration of Ni(II) in the residual solution using ICP-MS. Calculate the adsorption capacity of Ni(II) by the MS and MMCs based on Equation (1) [25]. The sorption experiments were replicated three times.

(1) $$q_{e}=\frac{(C_{0}-C_{e})\times V}{m}$$

where, *q_e_* represents the adsorption capacity (mg/g); *V* denotes the volume of the Ni(II)-containing wastewater (L); *C*_0_ and *C_e_* represent the nickel ion concentrations in the solution before and after adsorption (mg/L); and *m* refers to the mass of the MS and MMCs (g).

## 3. Results and Discussion

### 3.1. Characterization

**Figure 1:**
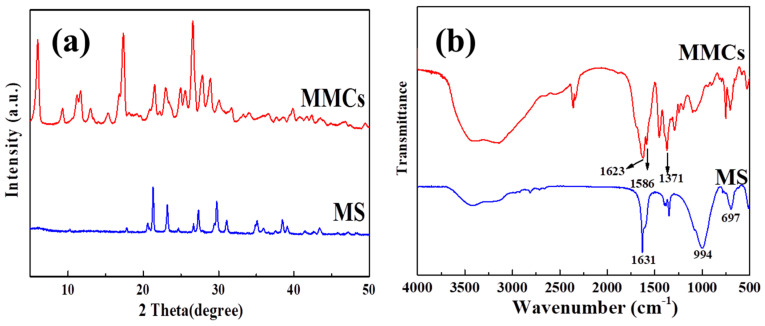
The XRD patterns (**a**) and FT−IR spectrometry (**b**) of the MS and MMCs.

The XRD spectra of MS and MMCs are depicted in Figure 1a. The graph illustrates that MS is composed of a well-oriented crystallized structure, with main characteristic peaks observed at 2θ = 10.2, 24.6, 27.5, and 30 degrees, which are attributed to the four-membered ring structure [17]. Upon introducing the ligand H_3_BDC into the MS to construct MOF layers, the material exhibits prominent MIL-96 characteristic peaks. Concurrently, some characteristic peaks of the MS still persist, indicating the successful preparation of the material. 

The FT-IR spectra of the MS and MMCs are shown in Figure 1b. The peak at 1631 cm^−1^ is associated with the bending vibration of the -OH group in the MS, while the peak at 997 cm^−1^ corresponds to the asymmetric stretching vibration characteristic peak of the Al-O tetrahedra, and the peak at 697 cm^−1^ is related to Si-O in the MS [18,26]. In the MMCs’ spectrum, the peaks at 1586 cm^−1^ and 1371 cm^−1^ are attributed to the asymmetric and symmetric stretching vibrations of carboxyl groups within the material [27]. Additionally, some characteristic peaks of the MS disappear or weaken, and some characteristic peaks of aluminum-based MOFs emerge, indicating the successful preparation of MMCs [20,28].

**Figure 2:**
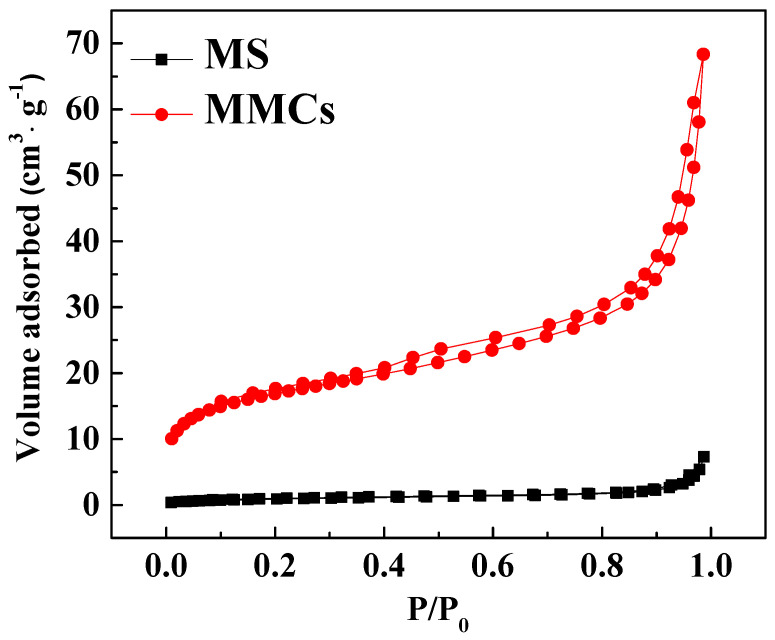
N_2_ adsorption−desorption isotherms of MS and MMCs.

The nitrogen adsorption–desorption isotherms of the MS and MMCs are shown in Figure 2, and Table 1 presents the structural characteristics data of nitrogen adsorption–desorption for the two materials. The results indicate that the nitrogen adsorption−desorption isotherm of the MS and MMCs belongs to the type IV curve, and the type IV isotherm is characterized by the accompaniment of the H_3_ hysteresis loop after capillary condensation, suggesting that mesoporous structures were formed on the MOF NFs. Compared to the MS, the specific surface area and pore volume of MMCs have increased, attributed to the formation of MOFs, which enhances the pore structure.

The specific surface area was calculated according to the BET equation, while the pore volume and pore diameter were determined using the BJH equation.

**Figure 3:**
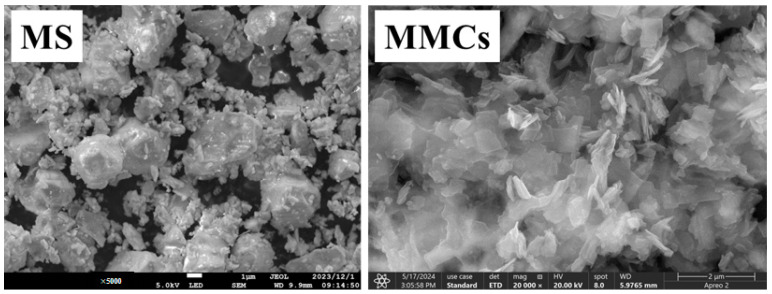
SEM images of MS and MMCs.

The SEM images of the MS and MMCs in Figure 3 reveal distinct morphological differences. The MS exhibits a spherical morphology, while the composite MMCs display a flaky structure, indicating that the formation of a multi-framework structure has influenced the physical appearance of the material.

### 3.2. Study on Adsorption Properties

#### 3.2.1. Effect of pH

**Figure 4:**
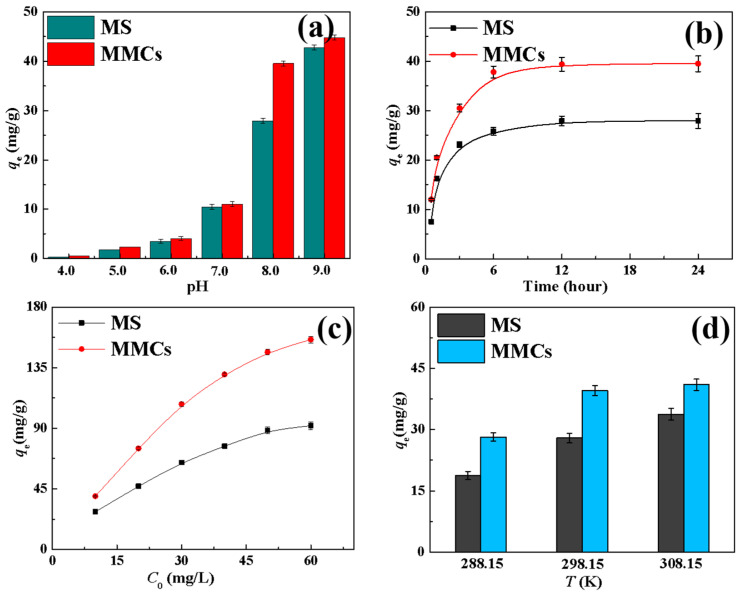
Effect of various parameters on adsorption efficiency of MS and MMCs for Ni(II): (**a**) pH; (**b**) contact time; (**c**) initial concentration of nickel ions; and (**d**) temperature.

From Figure 4a, it can be observed that when the pH was in the range of 4–7, the adsorption of Ni(II) by the MS and MMCs was below 10 mg/g. This can be attributed to the higher concentration of hydrogen ions in acidic conditions, leading to protonation of the adsorption sites on the materials. As a result, hydrogen ions competed with Ni(II) for adsorption, resulting in lower adsorption of Ni(II) by the MS and MMCs. As the pH increased, the impact of hydrogen ions gradually decreased, leading to a rapid increase in the adsorption of Ni(II) by the MS and MMCs. At pH 8.0, the adsorption capacities of Ni(II) by the MS and MMCs were 27.9 and 39.5 mg/g, respectively. At pH 9.0, due to the formation of Ni(OH)_2_ precipitation [3,29], the adsorption capacities of Ni(II) by the two materials reached 42.7 mg/g and 44.7 mg/g. Since Ni(OH)_2_ is the primary form of Ni(II) at this pH, further studies were conducted under pH = 8.0 conditions.

At pH 8.0, the adsorption capacity of Ni(II) by the MS was 27.9 mg/g, indicating that the prepared MS exhibited good adsorption performance. Upon the formation of MMCs by composite with MOFs, the adsorption capacity of Ni(II) further increased to 35.9 mg/g. This phenomenon suggests that the surface area of MMCs was enhanced after MOFs composite, thereby directly increasing the material’s capability to adsorb metal ions. Therefore, composite formation with MOFs is necessary and meaningful for enhancing the adsorption capacity of MSs.

#### 3.2.2. Effect of Contact Time

Figure 4a illustrates the impact of adsorption time on the Ni(II) adsorption performance of MSs and MMCs. It can be observed from the graph that the adsorption capacities of Ni(II) by the MS and MMCs both increase with prolonged adsorption time. In the initial stages of the adsorption reaction, the adsorption of Ni(II) rapidly increases with time but later slows down as a result of the decrease in Ni(II) concentration and diminishing reactive sites until reaching saturation. The adsorption capacities of Ni(II) by the MS and MMCs reach equilibrium at around 6 h, with adsorption amounts of 27.9 and 39.5 mg/g, respectively.

**Figure 5:**
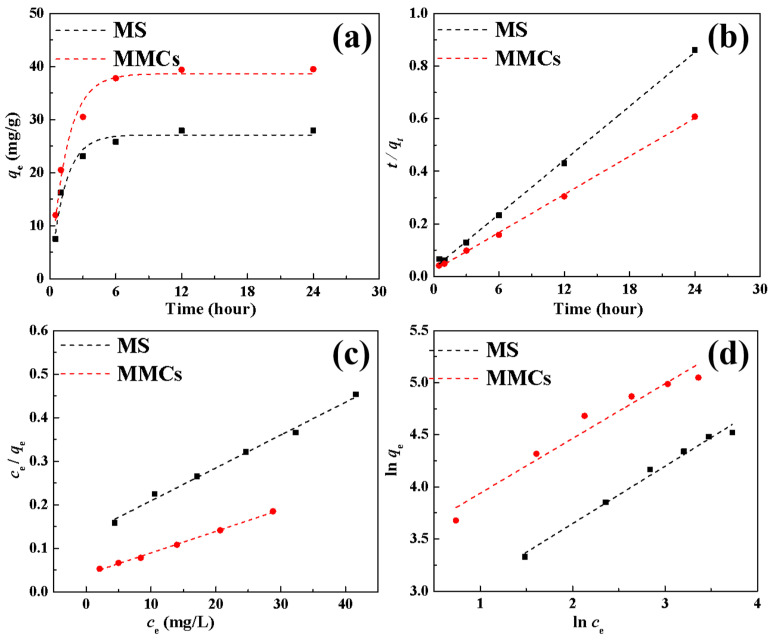
The kinetic and isotherm model for Ni(II) adsorption on the MS and MMCs. (**a**) PFO model, (**b**) PSO model, (**c**) Langmuir model, and (**d**) Freundlich model.

Figure 5a,b depict the fitting of pseudo-first-order (PFO, Equation (2)) and pseudo-second-order (PSO, Equation (3)) kinetic models [17,30] to the adsorption dynamics of Ni(II) by the MS and MMCs, along with the fitting parameters of the PFO and PSO models (Table 2). The two models are as follows:

(2) $$\ln(q_{e}-q_{t})=\ln q_{e}-k_{1}t$$

(3) $$\frac{t}{q_{t}}=\frac{1}{k_{2}{q_{e}}^{2}}+\frac{t}{q_{e}}$$

In this context, *q_t_* and *q_e_* represent the adsorption amount at time *t* and at equilibrium (mg/g), respectively. Additionally, *k*_1_ and *k*_2_ are the rate constants in the PFO and PSO models. 

Compared to the PFO model, the adsorption of Ni(II) by the MS and MMCs aligns well with the PSO model. Furthermore, the calculated *q_e,cal_* based on the PSO model is closer to the experimental values, indicating that the adsorption process of Ni(II) by the MS and MMCs still predominantly occurs through chemical adsorption.

#### 3.2.3. Effect of Initial Concentration of Ni(II)

The initial concentration plays a crucial role in determining the mass transfer efficiency of pollutants between the solid and liquid phases, making it another key factor influencing the adsorption capacity. By increasing the initial concentration of metal ions, the removal capacity of the adsorbent can be effectively enhanced. The effective removal of Ni(II) by the MS and MMCs follows this trend, as shown in Figure 4c. As the initial concentration of Ni(II) is raised from 10 mg/L to 60 mg/L, the adsorption capacity of the MS and MMCs for Ni(II) increases from 27.9 mg/g and 39.5 mg/g to 91.8 mg/g and 155.8 mg/g, respectively. This increase in adsorption is primarily attributed to the higher Ni(II) concentration, which boosts the concentration gradient between the adsorbate and the adsorbent, leading to improved mass transfer dynamics and facilitating more migration of Ni(II) to the adsorbent surface. Moreover, the higher Ni(II) concentration increases the likelihood of collision with active sites on the adsorbent. The isotherm fitting of Ni(II) adsorption on the MS and MMCs using Langmuir (Equation (4)) and Freundlich (Equation (5)) models [31,32] is displayed in Figure 5c,d, respectively, with the fitting parameters listed in Table 3. The Langmuir and Freundlich equations are as follows:

(4) $$\frac{c_{e}}{q_{e}}=\frac{c_{e}}{q_{m}}+\frac{1}{q_{m}k_{L}}$$

(5) $$\ln q_{e}=\ln K_{F}+\frac{1}{n}\ln c_{e}$$

where *C_e_* (mg/L) is the equilibrium Ni(II) concentration, *q_e_* and *q_m_* (mg g^−1^) are the equilibrium and maximum theoretical adsorption of Ni(II), respectively, *K_L_* (L mg^−1^) is the Langmuir adsorption equilibrium constant, and *K_F_* (mg g^−1^(L mg^−1^)^1/n^) and n are the Freundlich constants related to the adsorption capacity and adsorption strength, respectively.

It is evident that the adsorption of Ni(II) by the MS and MMCs conforms well to the Langmuir model, with higher R^2^ values (Table 3) indicating a monolayer adsorption process for Ni(II). The maximum adsorption capacities of Ni(II) by the MS and MMCs are 133.3 mg/g and 204.1 mg/g, respectively, aligning well with the experimental trends. Furthermore, the significantly higher adsorption capacity of MMCs compared to MSs indicates the successful modification of MSs. Table 4 compares the adsorption capacities of different adsorbents for Ni(II), revealing that MMCs demonstrate a significantly higher adsorption capacity for Ni(II), indicating their substantial potential in the separation of Ni(II). 

#### 3.2.4. Effect of Temperature

Figure 4d illustrates the impact of temperature on the adsorption of nickel ions by the MS and MMCs. It can be seen that as the temperature rises from 288 K to 308 K, the adsorption capacity of the MS and MMCs for nickel ions gradually increases. This indicates that the adsorption of nickel ions by both adsorbents is an endothermic process, and the increase in temperature promotes the adsorption of nickel ions by the MS and MMCs. To further investigate the adsorption mechanism of MSs and MMCs for nickel ions, the enthalpy change (Δ*H*^0^), entropy change (Δ*S*^0^), and Gibbs free energy (Δ*G*^0^) of the adsorption process of Ni(II) by the MS and MMCs were calculated according to Equations (6) and (7) [39]. The results are shown in Table 5. Since Δ*H*^0^ > 0 and Δ*G*^0^ < 0 for the adsorption of nickel ions by the MS and MMCs, it indicates that the adsorption of nickel ions by both materials is a spontaneous endothermic process.

(6) $$\Delta G^{0}=\Delta H^{0}-T\Delta S$$

(7) $$\ln K_{d}=\frac{\Delta S^{0}}{R}-\frac{\Delta H^{0}}{RT}$$

#### 3.2.5. Study on Cycle Stability

**Figure 6:**
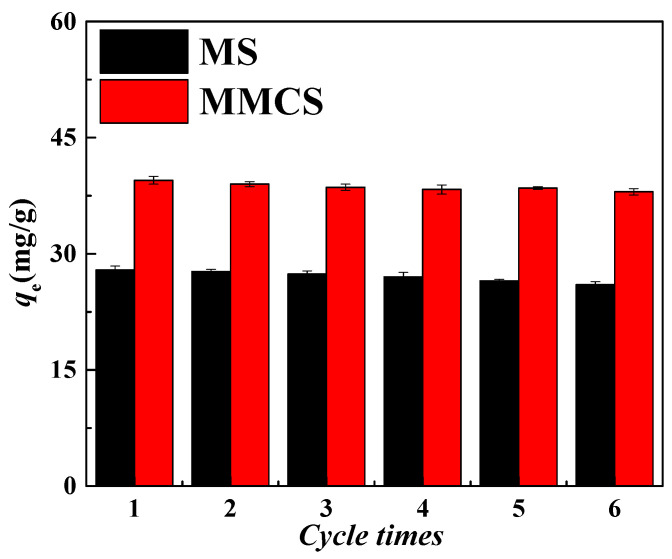
Study on cyclic stability of MS and MMCs.

The repeated use of adsorbent materials is an extremely important factor in determining their adsorption performance. The cyclic regeneration performance of MSs and MMCs was studied through adsorption–desorption cycles. After adsorption of Ni(II) by the two materials, they were repeatedly washed with 1.0 M hydrochloric acid to desorb Ni(II) until no Ni(II) was detected in the wash solution. Subsequently, the MS and MMCs were used again for adsorption experiments in the same concentration of Ni(II) solution. The adsorption and desorption cycles were repeated six times for the MS and MMCs, and the adsorption capacity for each cycle was recorded, as shown in Figure 6. After six adsorption–desorption cycles, the adsorption capacity of the MS and MMCs remained at over 90% of the original adsorption capacity. Therefore, the desorption process did not alter the structure and chemical properties of the MS and MMCs, indicating that they possess good stability and regeneration performance.

#### 3.2.6. Study on Adsorption Mechanism

**Figure 7:**
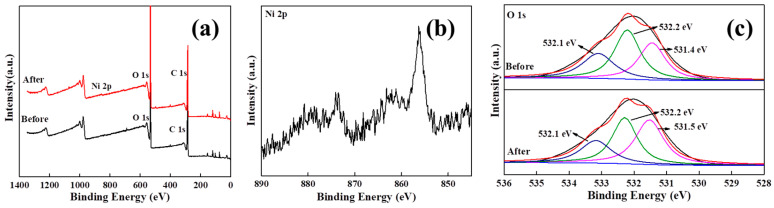
XPS spectra of full spectrum: (**a**) Ni 2p, (**b**) O 1s, and (**c**) before and after adsorption of MMCs.

To investigate the adsorption mechanism of nickel ions by MMCs, XPS scan spectroscopy analysis was conducted before and after the adsorption of nickel ions on MMCs, as shown in Figure 7. The XPS spectra of MMCs before and after nickel ion adsorption are shown in Figure 7a. From the spectra, a new peak with a binding energy of 856.2 eV appeared on the surface of MMCs, indicating the successful adsorption of nickel ions on the surface of MMCs [1,4]. The O 1s spectrum of MMCs (Figure 7c) shows three peaks at 531.4 eV, 532.2 eV, and 533.1 eV, corresponding to the oxygen atoms in Al-O_BTC_, Si-O-Si, and Si-O-H structures, respectively [6,8,40]. After the adsorption of nickel ions by MMCs, a chemical shift to the left occurred in the binding energy of Si-O-H, indicating a chemical transformation in the chemical form of oxygen atoms. This is due to the oxygen atoms providing lone pair electrons to the nickel ions to form coordination bonds.

## 4. Conclusions

In summary, in this study, MS-based MOF composite materials (MMCs) were prepared using H_3_BDC as a ligand. These materials were utilized for the separation of nickel ions, and the results indicated that adsorption equilibrium could be achieved after 6 h at pH 8, with equilibrium adsorption capacities of 27.9 mg/g and 39.5 mg/g for the MS and MMCs, respectively. The adsorption of nickel by the MS and MMCs followed the Langmuir model and pseudo-second-order kinetic model, with a theoretical maximum adsorption capacity of 133.3 mg/g and 204.1 mg/g. Furthermore, the adsorption capacity of nickel ions by MMCs was higher than that of the MS, indicating the successful preparation of the composite material. The adsorption process was mainly driven by a coordination mechanism. This study demonstrates that the utilization of MMCs for the adsorption and separation of nickel ions is effective and holds significant implications for the treatment and disposal of spent LIBs. Future research could further explore different combinations of ligands and matrices to enhance the adsorption performance of composite materials for nickel ions. Additionally, consideration could be given to optimizing the preparation process to improve the stability and regenerability of the composite material. Furthermore, exploring the application of this composite material for the adsorption and separation of other metal ions could expand its use in environmental remediation and resource recovery. Through ongoing research and improvements, the adsorption and separation performance of the composite material can be further enhanced, providing a more effective solution for the disposal of spent LIBs and related issues.

## Figures and Tables

**Table 1:** Pore structure parameters of MS and MMCs.

Sample	Specific Surface Area (m^2^/g)	Pore Volume (cm^3^/g)	Pore Diameter (nm)
MS	3.4	0.01	16.6
MMCs	65.0	0.09	11.5

**Table 2:** The kinetic model parameters for MS and MMCs adsorption on Ni(II).

Samples	PFO Model			PSO Model		
	*q_e_* (mg g^−1^)	*k*_1_ (min^−1^)	R^2^	*q_e_* (mg g^−1^)	*k*_2_ (g mg^−1^·min^−1^)	R^2^
MS	27.1	0.761	0.975	29.2	0.0369	0.998
MMCs	38.6	0.669	0.979	41.5	0.0252	0.999

**Table 3:** The isothermal model parameters of Ni(II) adsorption on the MS and MMCs.

Samples	Langmuir Model			Freundlich Model		
	q_m_ (mg g^−1^)	K_L_ (L mg^−1^)	R^2^	*K_F_* [(mg g^−1^)·(L mg^−1^)^1/n^]	n	R^2^
MS	133.3	0.056	0.993	12.8	1.82	0.988
MMCs	204.1	0.122	0.998	30.4	1.90	0.954

**Table 4:** A comparison of Ni(II) sorption from the literature using different adsorbents.

Adsorbent	Sorption Capacity (mg g^−1^)	Reference
MIIPs	18.5	[33]
Cloisite Na^+^	32.5	[34]
MgFeAlO_4_-NH_2_	201.6	[35]
MZ	0.025	[36]
ZrO(OH)_2_/VMT	90.2	[37]
Cell-g-NIPAM-co-GMA	74.7	[38]
MMCs	204.1	This Study

**Table 5:** The thermodynamic parameters of Ni(II)adsorption on the MS and MMCs.

Samples	T (K)	ln*K*	Δ*G*^0^ (kJ·mol^−1^)	Δ*S*^0^ (J·mol^−1^·K^−1^)	Δ*H*^0^ (kJ·mol^−1^)
MS	288	1.09	−2.5	53.6	12.9
	298	1.85	−3.1		
	308	2.34	−3.6		
MMCs	288	1.87	−1.4	125.6	34.8
	298	2.94	−2.6		
	308	3.13	−3.9		

## Data Availability

The data presented in this study are available upon request from the corresponding author.
